# The effect of hormone therapy on all-cause and cardiovascular mortality in women with chronic kidney disease: protocol for a systematic review and meta-analysis

**DOI:** 10.1186/s13643-015-0020-8

**Published:** 2015-04-08

**Authors:** Sharanya Ramesh, Michelle C Mann, Jayna M Holroyd-Leduc, Stephen B Wilton, Matthew T James, Ellen W Seely, Sofia B Ahmed

**Affiliations:** Faculty of Medicine, University of Calgary, 3330 Hospital Drive NW, Calgary, T2N 4N1 Alberta Canada; Libin Cardiovascular Institute of Alberta, 1403 29 Street NW, Calgary, T2N 2T9 Alberta Canada; Alberta Kidney Disease Network, 1403-29th St. NW, C210, Calgary, T2N 2T9 Alberta Canada; Division of Endocrinology, Diabetes and Hypertension, Department of Medicine, Brigham and Women’s Hospital, 75 Francis St., Boston, MA 02115 USA

**Keywords:** Hormone therapy, Chronic kidney disease, Cardiovascular mortality, All-cause mortality, Cardiovascular morbidity

## Abstract

**Background:**

Chronic kidney disease affects approximately one in ten North Americans and is associated with a high risk of cardiovascular disease. Chronic kidney disease in women is characterized by an abnormal sex hormone profile and low estradiol levels. Since low estradiol levels are associated with an increased cardiovascular risk in healthy women, our objective is to determine the effect of hormone therapy on all-cause mortality, cardiovascular mortality, and cardiovascular morbidity in women with chronic kidney disease.

**Methods/design:**

Studies examining hormone therapy for adult women with chronic kidney disease will be included. The primary outcome is all-cause or cardiovascular mortality and morbidity. We will search electronic bibliographic databases (MEDLINE, EMBASE, and the Cochrane Central Register of Controlled Trials (CENTRAL)) along with relevant conference proceedings, table of contents of journals, and review articles. Two investigators will independently screen identified abstracts and select observational cohort studies, case–control studies, and randomized controlled trials examining hormone therapy in women with chronic kidney disease. These investigators will also independently abstract data from relevant full-text journal articles and assess risk of bias. Where possible, these data will be summarized using pooled or combined estimates for the risk ratio or hazard ratio of all-cause mortality, cardiovascular mortality, and cardiovascular morbidity in women with chronic kidney disease with and without hormone therapy. A random effects model will be used, and meta-regression and subgroup analyses will be used to explore potential source of heterogeneity.

**Discussion:**

Given the high burden of cardiovascular disease in women with chronic kidney disease, this study will help guide clinical practice by summarizing current evidence on the use of hormone therapy for prevention of all-cause mortality, cardiovascular mortality, and cardiovascular morbidity in this population.

**Systematic review registration:**

The final protocol was registered with PROSPERO (CRD42014014566).

## Background

Chronic kidney disease (CKD) is a prevalent condition with a high burden of all-cause and cardiovascular (CV) morbidity and mortality [1,2]. Women with CKD have an abnormal sex hormone profile characterized by the lack of pulsatile gonadotropin-releasing hormone (GnRH) secretion and low estradiol levels [3,4]. As a low estrogen state is associated with increased CV risk [5], it may contribute to the increase in CV morbidity and mortality observed in women with CKD.

There is significant epidemiological evidence suggesting a cardioprotective function for ovarian sex hormones and, in particular, estradiol in women [6-12]. For example, the prevalence of cardiovascular disease (CVD) increases with age in men; however, women are relatively protected until menopause, after which time morbidity and mortality from CVD equal or exceed that of men [13]. Randomized control trials demonstrated no evidence of cardioprotection with hormone therapies [14-16]; however, later analysis of these studies have shown that the timing of hormone therapy initiation [17], route of administration of estrogen [18], and the type of estrogen and progesterone used [19] impact CV risk significantly.

In women with CKD, disruptions in the GnRH production lead to a hypoestrogenic state and cessation of menses and infertility, both of which return to normal levels with successful kidney transplants [20-23]. There is evidence to suggest that this loss of estradiol in women with CKD is associated with increased CV risk. In contrast to healthy women [24], women with end-stage kidney disease (ESKD) have similar overall mortality rates compared to age-matched men [25-27]**.** It is hypothesized that this increased risk may be due to the lower lifetime exposure to estradiol as a result of onset of kidney disease earlier in life [26]. In amenorrheic women with CKD, estrogen therapy has favorable effects on lipid profiles by decreasing LDL levels and increasing HDL levels [28-30].

Although a recent meta-analysis of postmenopausal hormone therapy in healthy postmenopausal women found no evidence of cardioprotection, the results varied among the included studies due to the different hormone regimens, the timing of hormone therapy initiation, and the type of estradiol and progestin used [19,31]. Additionally, it is important to note that 8 of the 13 trials included in the meta-analysis were examining the effect of hormone therapy as secondary, rather than primary, prevention in a high CV-risk population [31]. Whether these results are translatable to a high CV-risk population with CKD-mediated low levels of estradiol is unclear as the evidence has not been systematically summarized. Given the paucity of work on the therapeutic role of hormone therapy in the CKD population, we propose to complete a systematic review to determine the effect of hormone therapy on all-cause mortality, cardiovascular mortality, and cardiovascular morbidity in women with CKD. Additionally, given that the timing of the administration of hormone therapy (perimenopausal vs postmenopausal) influences risk, we aim to determine whether age, a surrogate for menopausal status, at administration of hormone treatment modifies outcomes in the CKD population [32].

## Methods

### Design

The systematic review protocol was designed based on the guidance from the Preferred Reporting Items for Systematic reviews and Meta-analyses Protocol (PRISMA-P) [33].

### Protocol

To determine the effect of hormone therapy on all-cause mortality, cardiovascular mortality, and cardiovascular morbidity (as defined by individual studies) in women with CKD, we developed a systematic review and meta-analysis protocol using the Cochrane guidelines for systematic review and meta-analysis [34]. The final protocol was registered with PROSPERO (CRD42014014566) [35]. The population, exposure, comparator, outcome, and study design (PECOD) utilized within this systematic review are presented in Table 1.

**Table 1:** **PECOD question for the systematic review**

**Population**	**Women with chronic kidney disease (as defined by eGFR or presence of kidney damage)**
Population	Women with chronic kidney disease (as defined by eGFR or presence of kidney damage)
Exposure	Hormone Therapy (HT) (all types of HT including estradiol alone or estradiol and progesterone)
Comparator	Placebo or No HT
Outcome	All-cause and cardiovascular mortality
Design	RCTs, cohort, and case–control studies

### Search strategy

#### Bibliographic databases

The online databases to be searched are MEDLINE (1950 to September 2014), EMBASE (1980 to September 2014), and the Cochrane Central Register of Controlled Trials (CENTRAL). The Medline search will be peer reviewed [36].

#### Other sources

Two individuals will search the reference list of relevant publications, abstracts of all major North American Nephrology and Cardiovascular meetings, (American Society of Nephrology, Canadian Society of Nephrology, American Heart Association Scientific Sessions and American College of Cardiology) between 2011 and 2014, and the tables of contents of major nephrology journals (Journal of American Society of Nephrology, Kidney International and American Journal of Kidney Diseases) and cardiology journals (Circulation, Journal of the American College of Cardiology) from January 2011 to September 2014. In order to get information about ongoing or unpublished studies, experts in the field will be contacted. Experts will be identified through the abstract review process.

#### Search limits

The search of online databases will include all languages and will not be restricted based on time period; however, it will be limited to studies on humans.

#### Search terms

Two separate searches will be conducted:

The first search will include the population and exposure terms of the PECOD question (Table 2):

**Table 2:** **Search strategy for the chronic kidney disease and hormone replacement therapy search**

	**Population and Exposure terms of the PECOD question**
1	Exp hormone replacement therapy/
2	Hormone replacement therap$.tw.
3	(HRT or ERT or ORT).ti,ab.
4	Exp estrogen replacement therapy/
5	(Hormone adj3 replacement adj3 therap$).mp.
6	(?Estrogen adj3 replacement adj3 therap$).mp.
7	?Estrogen replacement therap$.tw.
8	?Estradiol replacement therap$.tw.
9	Hormone therap$.tw.
10	Postmenopausal hormone therap$.tw.
11	Post-menopausal hormone therap$.tw.
12	1 or 2 or 3 or 4 or 5 or 6 or 7 or 8 or 9 or 10 or 11
13	Postmenopause.tw.
14	Exp postmenopause/
15	MENOPAUS$4.tw.
16	POSTMENOPAUS$4.tw.
17	POST-MENOPAUS$4.tw.
18	Exp perimenopause/
19	Perimenopaus$.tw.
20	Peri-menopaus$.tw.
21	Exp menopause/
22	?Estrogen.tw.
23	Exp estrogens/
24	?Estradiol.tw.
25	Exp estradiol/
26	22 or 23 or 24 or 25
27	or/13-21
28	26 and 27
29	12 or 28
30	Exp kidney failure/
31	Exp chronic kidney failure/
32	Exp renal insufficiency/
33	Exp renal insufficiency, chronic/
34	Exp chronic kidney disease/
35	Exp kidney disease/
36	Exp renal replacement therapy/
37	Exp uremia/
38	Exp hemodialysis/
39	Exp kidney transplantation/
40	(Haemodialys* or hemodialys*).tw.
41	Dialysis.tw.
42	Peritoneal dialysis.tw.
43	Exp peritoneal dialysis/
44	(CAPD or CCPD or APD).tw.
45	(Chronic renal or chronic kidney).tw.
46	Exp dialysis/
47	Ur?emi$.tw.
48	Kidney diseas*.tw.
49	Kidney failure.tw.
50	(H?emodia* or h?emofiltr*).tw.
51	(Pre-dialysis or predialysis).tw.
52	(Kidney transplant* or renal transplant*).tw.
53	(Kidney allograft* or renal allograft*).tw.
54	(Kidney graft* or renal graft*).tw.
55	(End-stage renal or end-stage kidney or endstage renal or endstage kidney).tw.
56	(ESRF or ESKF or ESRD or ESKD).tw.
57	(CKF or CKD or CRF or CRD).tw.
58	or/30-57
59	29 and 58
60	Limit 59 to animals
61	Limit 59 to (Animals and humans)
62	60 not 61
63	59 not 62

#### Chronic kidney disease

The first Boolean search will be done using the “OR” term to combine “exploded” MeSH subject headings and “text word” searches of the following:

“Renal insufficiency chronic”, “Kidney Failure”, “Kidney Diseases”, “Renal Replacement Therapy”, “Uremia”, “Dialysis”, “Hemodialysis”, “Hemofiltration”, “Peritoneal Dialysis”, “Predialysis”, “Kidney/Renal Transplant”, “Kidney/Renal Allograft”, “End Stage Renal/Kidney”, “Chronic Kidney Failure” or “Chronic Kidney Disease”

#### Hormone therapy

The second Boolean search will be done using the “OR” term to combine “exploded” MeSH subject headings and “text word” searches for the following:

“Hormone Therapy”, “Hormone Replacement Therapy”, “Estrogen Replacement Therapy”, “Estrogen”, “Estradiol”, “Estriol”, “Conjugated Equine Estrogen”, “Progesterone”, “Progestin”, “Medroxyprogesterone acetate” or “Dydrogesterone”

The above two will be combined using the “AND term.

The second search will include the exposure, outcome, and design terms of the PECOD question (Table 3). The purpose of this search is to identify randomized controlled trials that included subjects with CKD and conducted subgroup analysis for these subjects.

**Table 3:** **Search strategy for the cardiovascular disease and hormone replacement therapy and randomized control trial search**

	**Exposure, outcome, and design terms of the PECOD question**
1	Exp cardiovascular diseases/
2	Cardio*.tw.
3	Cardia*.tw.
4	Heart*.tw.
5	Coronary.tw.
6	Angina*.tw.
7	Ventric*.tw.
8	Myocard*.tw.
9	Pericard*.tw.
10	Isch?em*.tw.
11	Emboli*.tw.
12	Arrhythmi*.tw.
13	Thrombo*.tw.
14	Atrial fibrillat*.tw.
15	Tachycardi*.tw.
16	Endocardi*.tw.
17	(Sick adj sinus).tw.
18	Exp Stroke/
19	(Stroke or strokes).tw.
20	Cerebrovasc*.tw.
21	Cerebral vascular.tw.
22	Apoplexy.tw.
23	(Brain adj2 accident*).tw.
24	((Brain* or cerebral or lacunar) adj2 infarct*).tw.
25	Exp hypertension/
26	(Hypertension$ or hypertensive$).tw.
27	Peripheral arter* disease*.tw.
28	((High or increased or elevated) adj2 blood pressure).tw.
29	Exp hyperlipidemias/
30	Hyperlipid*.tw.
31	Hypercholesterol*.tw.
32	Hyperlipoprotein?emia*.tw.
33	Hypertriglycerid?emia*.tw.
34	Exp arteriosclerosis/
35	Exp cholesterol/
36	Cholesterol.tw.
37	‘Coronary risk factor*’.tw.
38	Blood pressure/
39	Blood pressure.tw.
40	or/1-39
41	Exp hormone replacement therapy/
42	Hormone replacement therap$.tw.
43	(HRT or ERT or ORT).ti,ab.
44	Exp estrogen replacement therapy/
45	(Hormone adj3 replacement adj3 therap$).mp.
46	(?Estrogen adj3 replacement adj3 therap$).mp.
47	?Estrogen replacement therap$.tw.
48	?Estradiol replacement therap$.tw.
49	Hormone therap$.tw.
50	Postmenopausal hormone therap$.tw.
51	Post-menopausal hormone therap$.tw.
52	41 or 42 or 43 or 44 or 45 or 46 or 47 or 48 or 49 or 50 or 51
53	Postmenopause.tw.
54	Exp postmenopause/
55	MENOPAUS$4.tw.
56	POSTMENOPAUS$4.tw.
57	POST-MENOPAUS$4.tw.
58	Exp menopause/
59	Exp perimenopause/
60	Peri-menopaus$.tw.
61	Perimenopaus$.tw.
62	?estrogen.tw.
63	Exp estrogens/
64	?estradiol.tw.
65	Exp estradiol/
66	62 or 63 or 64 or 65
67	or/53-61
68	66 and 67
69	52 or 68
70	Randomized controlled trial.pt.
71	Controlled clinical trial.pt.
72	Randomized.ab.
73	Placebo.ab.
74	Drug therapy.fs.
75	Randomly.ab.
76	Trial.ab.
77	Groups.ab.
78	70 or 71 or 72 or 73 or 74 or 75 or 76 or 77
79	Limit 78 to animals
80	Limit 78 to (animals and humans)
81	79 not 80
82	78 not 81
83	Exp mortality/
84	Exp survival rate/
85	All cause mortality.tw.
86	Mortality.tw.
87	Cause of death.tw.
88	Exp death/
89	Death.tw.
90	Exp “Cause of death”/
91	Survival rate.tw.
92	83 or 84 or 85 or 86 or 87 or 88 or 89 or 90 or 91
93	40 or 92
94	69 and 82 and 93
95	Fabry.tw.
96	Fabry’s.tw.
97	95 or 96
98	Growth hormone.tw.
99	97 or 98
100	94 not 99

#### Hormone therapy

The first Boolean search will be done using the “OR” term to combine “exploded” MeSH subject headings and “text word” searches for the following:

“Hormone therapy”, “Hormone Replacement Therapy”, “Estrogen Replacement Therapy”, “Estrogen”, “Estradiol”, “Estriol”, “Conjugated Equine Estrogen”, “Progesterone”, “Progestin”, “Medroxyprogesterone acetate”, or “Dydrogesterone”

#### Cardiovascular disease and all-cause mortality

The second Boolean search will be done using the “OR” term to combine “exploded” MeSH subject headings and “text word” searches for the following:

*“*Cardiovascular Diseases”, “Stroke”, “Hypertension”, “Hyperlipidemias”, “Arteriosclerosis”, “Cholesterol” “Blood Pressure”, “Coronary Heart Disease”, “Mortality”, “Survival Rate”, “All Cause Mortality”, “Cause of Death”, or “Death”

#### Randomized control trial

The third Boolean search will be done using the “OR” term to combine “exploded” MeSH subject headings and “text word” searches for the following:

“Randomized Controlled Trial”, “Controlled Clinical Trial”, “Placebo”, or “Drug Therapy”

The above three will be combined using the “AND” term. Additionally, articles with the terms “Fabry’s Disease” and “Growth Hormone Therapy” in the title or abstract will be excluded.

### Identification of articles for eligibility

#### Initial screening

After a calibration exercise in order to identify any systematic discrepancies in classification of abstracts between the two reviewers, screening will be conducted by two independent reviewers to determine articles eligible for the systematic review. An initial screen of identified abstracts from the databases and other sources will be performed to identify relevant articles for a full-text review. An abstract from the search will be considered for a full-text review if it meets the PECOD question criteria. This initial screening is intended to be sensitive and broadly capture any relevant articles studying hormone therapy in women with chronic kidney disease. In order to provide a comprehensive systematic review of the topic, the search and eligibility will not be restricted by a specific geographic region or publication year.

The decision of each reviewer for each abstract will be recorded, and any disagreement between reviewers will be resolved by consensus at all stages of the screening process. Authors will be contacted for missing data or clarification. For the initial screening search, if there is a lack of consensus among the reviewers and one of the reviewers feels that a full-text review is required, the article in question will qualify for a full-text review.

#### Full-text review

The same two individuals will scan full-text articles, and those that meet the following criteria will be considered for the systematic review:

- Population: The study includes women with chronic kidney disease
- Exposure: Hormone therapy
- Comparator: Placebo or no hormone therapy
- Outcome: Hazards or risks of all-cause and cardiovascular mortality
- Study Design: RCT, cohort study, or case–control study

This will capture trials and cohort and case–control studies that compare hormone therapy to placebo or no hormone therapy on the risks or hazards of all-cause mortality, cardiovascular mortality, and cardiovascular morbidity in women with CKD for potential meta-analysis. The decision of each reviewer will be documented, and the agreement between the reviewers will be quantified by the Kappa statistic. Any disagreement between reviewers will be resolved by consensus.

### Data extraction

A data extraction form will be developed to record and analyze information from each of the identified studies. The data will be extracted and verified by both reviewers in order to decrease reviewer error and bias. Data will be abstracted on study identification (authors, year of publication, and location of centers), characteristics of study design (sample size, inclusion and exclusion criteria, length of follow-up, type of hormone therapy, dosage of hormone therapy), characteristics of study participants (mean/median age, presence of comorbidities, stage of CKD, mean glomerular filtration rate (GFR), duration of CKD, time on dialysis), and outcome variable of interest (risk of all-cause mortality, cardiovascular mortality, and cardiovascular morbidity). Outcomes of RCTs and observational studies will be recorded separately. The data will be organized and managed in Endnote (version X7, Thomson Reuters, New York, NY) and Excel (version 2007, Microsoft Corporation, Redmond, WA).

### Risk of bias assessment

Each study will be appraised for quality and risk of bias by each reviewer using the Cochrane risk of bias tool for randomized controlled trials for RCTs or the Cochrane risk of bias tool for cohort studies [37]. Additionally, a Jadad score will be calculated for each trial [38]. This will be used to determine the strength of the body of evidence.

### Data synthesis and analysis

Data will be presented on the number and characteristic of articles identified for the initial screening and full-text review. The number of identified studies that were included and excluded (with reasons for exclusions) will be presented using the PRISMA flow chart [39].

The primary outcome of the risk ratio of all-cause mortality, cardiovascular mortality, and cardiovascular morbidity with hormone therapy compared to placebo or no hormone therapy will be reported from a potential meta-analysis of the RCTs and observational studies. If only the hazard ratio is presented, the number of events in each group will be abstracted from the study (if possible) and a risk ratio will be presented. If the above is not possible, we will present both risk ratio and hazard ratio estimates separately. The estimates will be presented separately for RCT and observational studies. As we anticipate the studies to be inherently different due to types of hormone therapy, dose regimen, etc., we will use the random effects model to pool estimates. Furthermore, statistical heterogeneity will be assessed using the Cochrane Q statistic and *I*^*2*^ statistic. If there is significant statistical heterogeneity, univariate meta-regression analysis will be done to assess the effects of the following variables on risk estimates: mean age, diabetes, cardiac history, comorbidities, mean estimated glomerular filtration rate, CKD stage, and route of administration of hormone therapy. Evidence of publication bias will be determined through visual inspection of funnel plots and Begg’s rank correlation test for asymmetry. All analysis will be performed using STATA (version 12, StataCorp LP, College Station, TX). If meta-analysis is not possible, we will present the results as a systematic review.

### Limitations

Although this methodology had been designed to minimize bias, we anticipate some limitations with this review. First, we anticipate a difference in the formulation and dosage of hormone therapy in different studies. Restriction of the inclusion criteria to a specific formulation or dosage of hormone therapy would likely result in a paucity of data. Therefore, in order to control for any variations in outcomes due to differences in type and dose of hormone therapy in the included studies, we will perform subgroup analyses based on the type and doses of hormone therapy used to understand their contribution to heterogeneity.

Second, our results may be confounded by the lack of study-level data on the prevalence of important comorbidities such as diabetes and hypertension. Due to this, our results would have to be interpreted with caution; however, we will attempt to provide sufficient evidence to help determine the clinical significance of the presented pooled estimates by conducting stratified analyses when possible.

## Discussion

In summary, this systematic review has the potential to have significant clinical implications. If a cardioprotective role of hormone therapy is identified, then this could provide a rationale for large multicenter RCTs to examine estrogen replacement as a therapy to mitigate the high CV risk in women with CKD. Conversely, if the cardioprotective effects of hormone therapy cannot be established, this evidence would be useful in enhancing both patients’ and physicians’ understanding of the limitations of this treatment.

To ensure that our findings have clinical impact on both patients and the physicians who care for them, we will disseminate our findings broadly by presenting at scientific conferences, publishing in a peer-reviewed journal as well as newsletters and websites of interested organizations, and conducting meetings with key stakeholders.

## Abbreviations

CV: Cardiovascular
CVD: Cardiovascular disease
eGFR: Estimated glomerular filtration rate
